# Complementary Parts of Disease

**Published:** 1876-11

**Authors:** Alex. McBride

**Affiliations:** Berea, Ohio


### SOUTHERN MEDICAL RECORD.

VOL. VI. NOVEMBER, 1876. NO. 11.

### Selections.

### COMPLEMENTARY PARTS OF DISEASE.

BY ALEX. McBRIDE, M.D., BEREA, OHIO.*

We have no substantial evidence that any new disease or feature of disease has been developed during historic ages; but we frequently find manifestations or facts of disease which have been disregarded or forgotten.

The materials of medical knowledge are so vast, and their manifestations so diversified and separated by such wide intervals, that the human mind, in grasping and striving for the present, actually forgets or ignores the past. My mission to-day is to remind the profession of some facts of disease which have long been forgotten or overlooked. I propose to call the attention of the profession to the fact that by carefully observing symptoms, apparently innocent, we can sometimes be prepared to meet those which are grave or serious.

I propose to treat of *complementary* parts of disease—those symptoms and signs which are sure precursors of something to come, or which may with certainty be expected to follow that which has already appeared. I do not propose to treat extensively of symtomatology, but to point out the phases of some of those diseases whose different parts or stages are so far sep–

* Read before the Cuyahoga County Medical Society, at Cleveland, December, 1874.

arated by time and contrariety of feature, as to appear to be different diseases, or to have no connection with each other.

When Dewees, a man for whom I have great veneration, wrote that the non-appearance or stoppage of the lochia, at an early stage of the puerperal state, was, *per se*, no cause for apprehension or alarm, he wrote perhaps what was true in a strict sense; but, nevertheless, it must have misled many. For in a practice of almost thirty years I have seen no case of such stoppage but what was soon followed by inflammatory symptoms. It would have been true if he said such stoppage should always, as a matter of safety, be regarded as the precursor of a grave inflammatory disease. He would thus have prepared the student for the exercise of the high attribute of a physician—caution. The fact is that stoppage of the lochia is primary or initial, and puerperal fever is complementary, of a grave disease. It may be objected to this illustration that puerperal inflammation is said to occur without stoppage of the lochia; but if such be the fact, it proves an exception to which all the rules of disease are liable.

A more illustrative case is that of cholera infantum. The physician's attention is called to a child with an extensive display cf heat-rash. He advises—"Let the child be dressed in light clothes, and well aired." Some days, or it may be weeks, later he is called to see the same child, and finds it pale and shrunken, the eyes sunken, and with a copious, serous diarrhœa and vomiting. The case is cholera infantum. Now, it does not happen that all cases, or nearly all, of *lichen tropicus* are followed by cholera infantum, no more than it happens that all cases of measles are followed by pneumonia; but it probably happens in all cases of sudden retrocession of the rash. Neither does it happen in all cases of cholera infantum that we can demonstrate the pre-existence of heat-rash; but it is a general fact that these conditions bear to each other the relation of initial and complementary parts of the same disease.

Now, we take a case of greater range of sequences. A woman complains that she has paroxysms of palpatation of the heart and dyspnœa, coming on suddenly and without warning; diz ziness and general discomfort when she knits or sews; belching; flatulence; rumbling of the bowels; a pain near the apex of the heart; pain at the middle of the left arm; hysterical convulsions, and numerous other distresses, which she will talk eloquently upon for an hour. Then, added to all this, in some cases, she has has a troublesome cough and soreness all over the chest; but notwithsanding she is supposed to have consumption, she has lost no flesh. When the doctor presses his thumb on the cervical and dorsal vertebræ, and finds great tenderness, she is surprised—she did not know it before. Many intelligent physicians understand this case, but there are many thousands who do not: I have known respectable and educated doctors to treat such cases for consumption. I have known others to treat for spinal disease; and the fact that cauterizing the spine relieved more or less all the symptoms, encouraged them to persevere. Valerian and a variety of stomachics are used; and for the pain in the side, blue-pill is sometimes used, which makes matters worse. In the entire range of disease, there is perhaps not another instance where such an extensive train of unhappy symptoms results from a cause so remote and apparently insignificant in the beginning. The woman is astonished and incredulous when told that this entire train of unhappy consequences has resulted from leucorrhœa, which had its beginning some years previous. But this is the fact; and to this there may have been added displacement of the uterus. Some will object to this explanation, and say the spinal irritation was the first link in the series of sequences. But of this view I have seen no proof. I know this—that curing the leucorrhœa and malpositions of the uterus, if any exis., cures the whole series of reflex ailments. How the morbid condition of the genital system causes spinal irritation is not clear, but the cause of the reflex symptoms which follow this is not so obscure. This I take to be a good illustration of complementary parts of disease.

A woman, near middle age, complains of aphonia; of being at intervals suddenly overcome with loss of strength; of periods of sweating; urine pale, and sometimes discharged very copiously; a general loss or lacking of strength. These symptoms are not constant, but frequently repeated, without any apparent provocation. A proper examination will reveal a large, flabby uterus. Such cases are apt to undergo treatment for whatever disease is in vogue at the time. It makes a very good case to treat for oxaluria. The case is relapsed uterus, and a proper use of ergot will cure it promptly. Iron may follow.

A woman presented herself, with an obstinate eczema covering one side of the face. I was so strongly impressed with the belief that such eruptions were complementary of some form of internal disease that I investigated. I found that she had severe dysmenorrhœa. Further research proved that there was narrowing of the cervix, and great irritability or cervicitis. The proper treatment of these latter conditions cured her face, and the cure was permanent. It is not claimed that these connections are constant; nothing is constant in disease. With our greatest research, we can arrive only at general facts.

In this connection, it may be remarked, as further illustrative, that endometritis is sometimes accompanied with a pain in the hip, which has been mistaken for coxalgia, and treated accordingly.

A patient applies, complaining of headache, more or less severe. There are no other symptoms. He calls from day to day; the pain is not intermittent; there are no collateral symptoms to indicate a fever; no evidences of gastric derangement; it is simply a headache. We may suppress it with aconite for a while, but quinine will not remove it. Dyspepsia or indigestion is suggested, but we find neither. Now this condition is initial to something which is to follow.

Experience has taught me, in a case of this kind, to look with confidence for an eruption of some sort; it may be of tertiary syphilis, in the form of nodes, caries, or any other form. It may be a series of boils or carbuncles. After one or the other of these appearances, the headache disappears. Syphilis may have laid dormant for many years, but when it breaks out, headache will precede it.

There is much reason for the belief that most of the cutaneous eruptions are complementary of internal disease—in fact, all except those which are *contageous*.

Take for an example, acne. We will not be particular which form, but will say the indurata and rosacea. Although it is not difficult to cause its temporary disappearance, no amount of local treatment will cure it. It is generally supposed to be connected with sexual irregularity of some kind. I am convinced that it has another cause. I find that all those persons, of either sex, who have acne badly, are great eaters—ravenous eaters. I never interrogated one closely, who did not confess that his appetite was nearly uncontrollable. This fact points to the stomach and duodenal portion of the bowel as the seat of the disease. Let these localities be relieved by proper evacuants, and kept relieved by temperance, and the trouble is relieved.*

Herpes zoster, although obscure as to its connections, is very clearly the complement of something interal.

Rubeola and scarlatina may be considered in connection. Every physician of several years' general practice, must have seen cases or an epidemic in which there was a mild pneumonia or pleuro-pneumonia, or perhaps bronchitis, or all three, accompanied by a rash resembling scarlatina and measles. Also a simlar epidemic, or sporadic cases, of a similar rash, with a considerable degree of inflammation about the throat, usually including the tonsils. The point to which I invite your attention in these cases is that these inflammations are seldom very severe when the rash co-exists. Physicians are generally in doubt as to whether these cases are scarlatina or not. For their safe treatment, little else is necessary than to favor the continuance of the rash. The rash and the phlegmasiæ are complementary of each other, and together constitute one disease.

Whatever the last-mentioned cases should be called, they hardly differ, except in degree, from their graver prototypes, scarlatina and reubeola; for the eruption of measles is but the completion of a disease which was originally a slight congestion of the lungs, or more especially of the bronchinal lining, and the

*It is nearly impossible to carry out these indications, for the reason that a ravenous appetite cannot be controlled. Arsenic is the medicinal remedy. It seems to be generally supposed that arsenic produces its alterative effect directly on the skin. From my experience in treating chronic diarrhœa with arsenic with great success, I am of the opinion that this remedy cures cutaneous diseases by curing the internal diseases upon which they depend.

eruption of scarlatina bears a similar relation to the region of the throat—so that when either of these retrocede, they merely resume their first position. (These diseases might, with propriety, be called metastatic diseases.)

These facts should call attention strongly to consider whether pure pneumonia should not have as its complement a rash. But without any theory of the kind, men always have, in severe cases of pneumonia, adopted a practice based upon such a theory. For what do we use blisters, sinapisms, and similar appliances, but to produce revulsion, by creating a rash or vesication, thus restoring the balance between initial and complementary parts?

Now let us consider that class of eruptions called tinea capitis, which occurs in children, for the term embraces at least both vesicular and pustular eruptions. From this we will exclude favus, which is topical and contagious. Now, what physician of experience dare attempt to suppress this disease by drying it up with no matter what remedy? He would expect, with confidence, to find soon after one or other of those forms of disease which we call hydrocephalus encephalitis, cerebritis, meningitis and the like.

On the other hand, what has been the most successful means of treating this last-named class of diseases? As a general rule, we expect patients thus affected to die. Sometimes, however, by the use of powerful alteratives they are saved, but almost never by the use of cold applications. But if the doctor has boldness enough to apply a blister nearly all over the scalp, he will have greater success in saving his patient. This every man knows who has tried it, but it is not done often because it looks so *terrible* in the eyes of the uninformed. Why does this apparently severe means save the patient? In my opinion, because it restores the balance of power—the other part of the disease—the complementary part. And here I will depart somewhat from my plan, and indicate a special method of treatment. It is not necessary to shave the scalp and apply a plaster. Cover the scalp with cantharidized oil, or tincture of cantharides, and then cover with oiled silk, a greased cloth, or green leaves, and you will have blister enough.* If the patient survives, you can remove the hair afterward, if necessary; and it will not look so bad when the patient is recovering. Some may think this case too strongly put, but I think not; and, if correct, it is one of the best illustrations of complementary parts, or, as some might prefer the expression, "transferable diseases."

But nothing can more strongly illustrate the antagonism between internal and external disease than tertiary syphilis. When there are copious manifestations of the disease on the skin and subcutaneous tissue, the bones and other internal parts almost wholly escape. The most deplorable case I ever saw was one in which there had been no external manifestations of any kind. Hence the excellence of iodide of potassium and guaiac, and other medicines which determine to the surface.

Rheumatism is a disease proper to be considered in this connection. This disease presents four parts which are worthy of consideration: 1. Arthritic and muscular or fibrous rheumatism; 2. Endocarditis, or cardiac rheumatism; 3. Symptomatic phrenitis; 4. Cutaneous eruptions, which may be vesicular, or a punctate rash resembling scarlatina. I do not know how many have observed this latter appearance, but I have had it under observation for several years.

General rheumatism and endocarditis are one and the same disease, and may co-exist, but it is more common to find the cardiac complication following the disappearance of the arthritic disease. When they co-exist, the cardiac complication is less grave than when it exists primarily, or follows the disappearance of the arthritic form. Hence the danger of suppressing the arthritic disease by ordinary local applications; for, inasmuch as they cause no elimination, the elements of the disease remain in the body, and are very liable to locate on the endocardium. The frenzy which is liable to follow is not disease, but a symptom. I remember hearing of metastasis of rheumatism to the brain;

*It may not be generally known that a blister can always be promptly raised by a mixture of one part of tincture of cantharides with one or two parts of any kind of oleaginous liniment, such as camphorated oil and tincture of cantharides, equal parts; apply freely on a linen rag and cover with oiled silk or green leaves. This saves all the *horrid* appearance of a blister plaster. It will generally answer the purpose sufficiently well to apply the plaster or blistering lotion about 2x5 inches down the nape of the neck.

and it has been my fortune twice during my life to correct reputable physicians who were vigorously treating for phrenitic cases which they should have treated for endocarditis. But the point to which I wish to call strongly your attention is the eruption which many times accompanies rheumatism. As before stated, the eruption is sometimes vesicular, and at other times a punctate rash, occuring on no particular part of the body, and seldom or never very copious. I have not yet derived anything positive from this eruption, except that the violence of the disease appears to be moderated by its irruption. Is not this rash or eruption an effort at something compensatory or complementary? The effects of certain kinds of treatment seem to indicate that it is. Now, there are three modes of treating rheumatism. These are the *eliminative*,* the *abortive*, and the *derivative* or *compensative*. Of the eliminative, I shall say nothing here. The abortive is applicable early in the disease, and the remedies are quinine and opium, either or both. The effect of opium, when used with sufficient boldness in the onset of the disease, is to arrest the pain, produce sweating and an eruption similar to urticaria, or, if no eruption, severe itching all over the body; and after this occurs, the general symptoms soon abate. The action of quinine is less marked and less effective.

The derivative or compensative method is by blistering. We have, of late, testimony from several sources that rheumatism, after having endured for a considerable time, has been rapidly cured by blistering. The blisters are drawn upon the chief seat or seats of the disease; but I am of the opinion that it is not very important to what part of the body they are applied. The cure, in this case, can hardly be owing to the evacuation by the blistered surface, and the amount of evacuation by the kidneys is not large. The revulsive action of a blister is considerable, it is true; but are all these sufficient to cure an established obstinate disease? It is my opinion, judging from analogy, that the cure results from the restoration of the lost element—the complementary part.

Before dismissing the subject, I will present one more view of

*The method by alkalies or acids, or both, I include among the eliminative, for they act as diuretics.

the matter. Nearly twenty-five years ago I treated the case of a boy who was slowly dying with softening of the heart. It was a case of cyanosis, the boy having been feeble from birth; and he was now twelve years old. During his final decline of about three months, he was freqnently seized with sharp pains in different parts of the body. The seizure and location of these pains did not appear to be governed by any rule. The pain would be in a finger, the face, jaw-bone, or in the intercostal muscles, or anywhere as it might happen. They were generally of brief duration, though sometimes lasting for hours. They were what I may call small, sharp pains. After the boy died, the autopsy showed softening of the heart and nonclosure of the foramen ovale. Shortly after this I attended a young woman during her decline with valvular disease. She, during many weeks, had similar pains, sometimes of great severity. The autopsy showed a case of literal "breaking of the heart strings"—the poetic expression verified. The chordæ tendinæ of the mitral valve had parted, and the valve had for weeks fluttered to and fro through the orifice. Subsequent observation has shown me that there is a nearly constant relation between cardiac disease and fugitive acute pains in various parts of the body. So much so, that for many years it has been my habit when a patient complains of the frequent recurrence of flying, lancinating pains, to look for disease of the heart; and I am seldom or never mistaken, except in the case which I shall presently mention. It appears to make little or no difference what the character of the cardiac disease is, whether valvular, pure hypertophy, dilation or softening. So much have I been guided by these pains, that, in the case of a young girl who was seized rather suddenly with pain of great severity in the eyeball or orbit, failing to perceive any other cause, I examined the heart, and readily found the valvular action very much disordered. The pain was soon relieved by morphine, but it was some years before valvular action was perfect.

The exception above alluded to is certain cases of dyspepsia. Some cases of dyspepsia give rise to the same or similar kind of pains, but of less severity. But in all such cases it will be found that the heart is functionally disordered. From the consideration, then, of both classes of cases, we derive this maxim, that a continuous recurrence of fugitive lancinating pains in various parts of the body (chiefly in the fibrous tissues) is indicative of cardiac disease—organic generally, but sometimes functional; or, to express the same concisely, the kind of pain above described are *complementary* of cardiac disease.

The complementary feature of disease might be treated at greater length, and its application shown in many cases.—*Lancet and Observer.*

